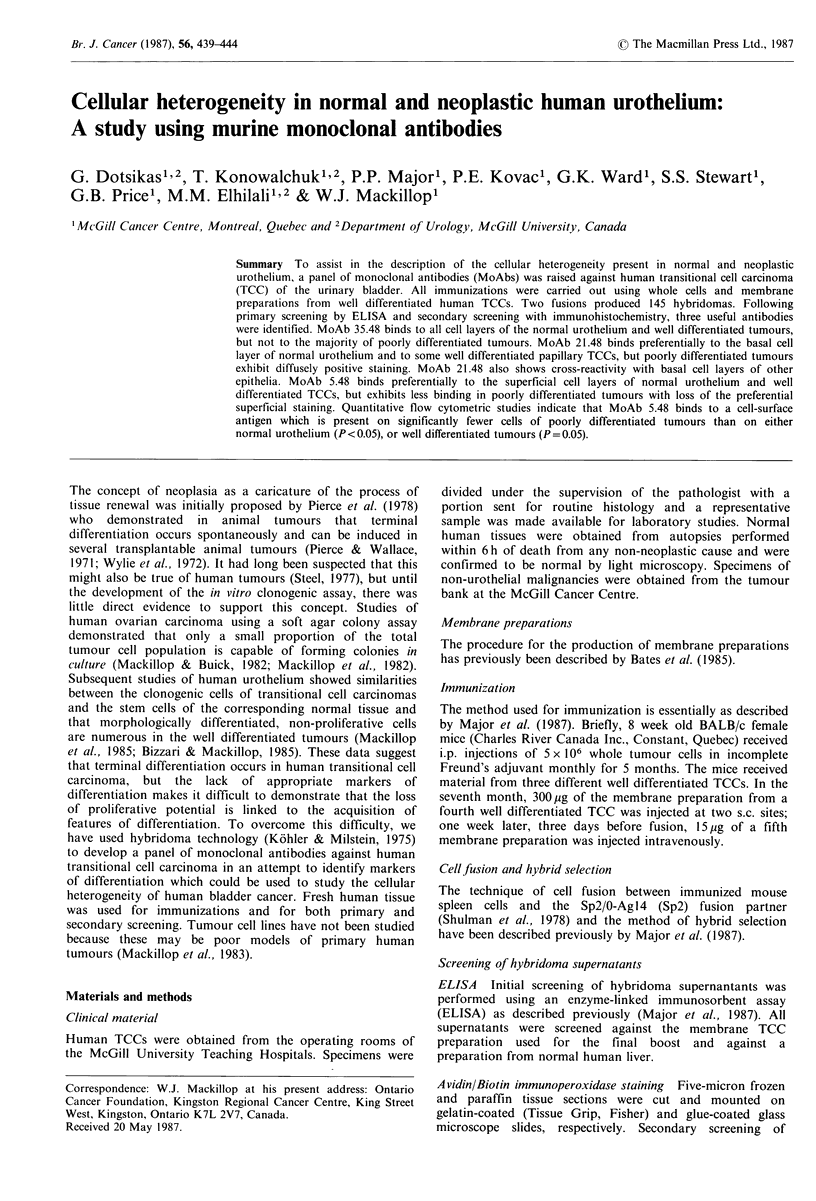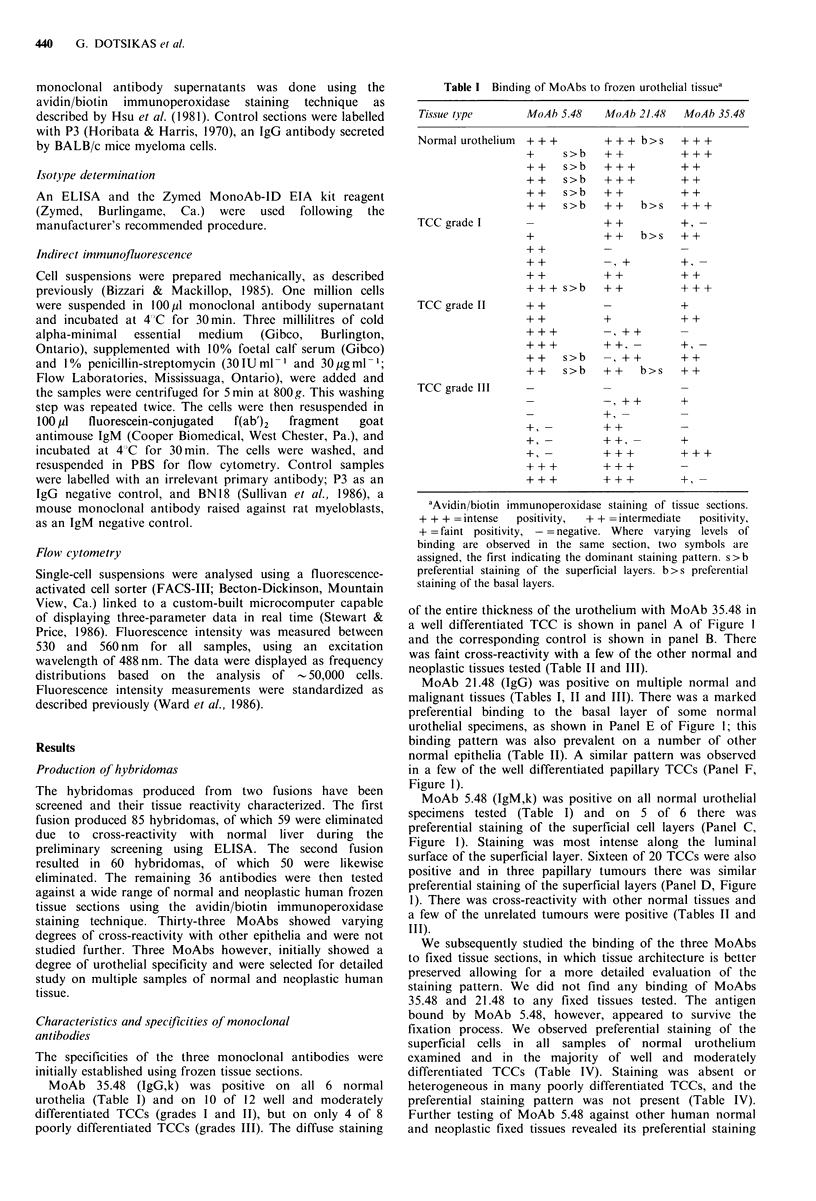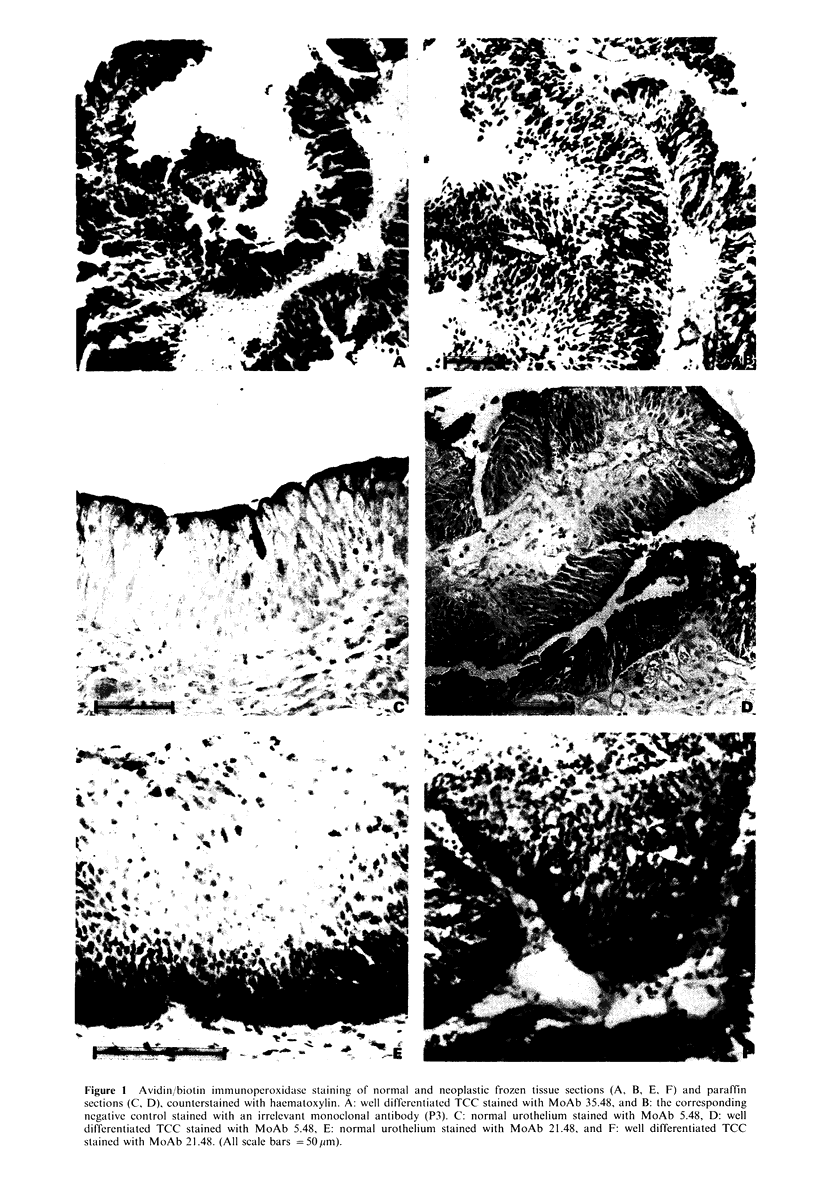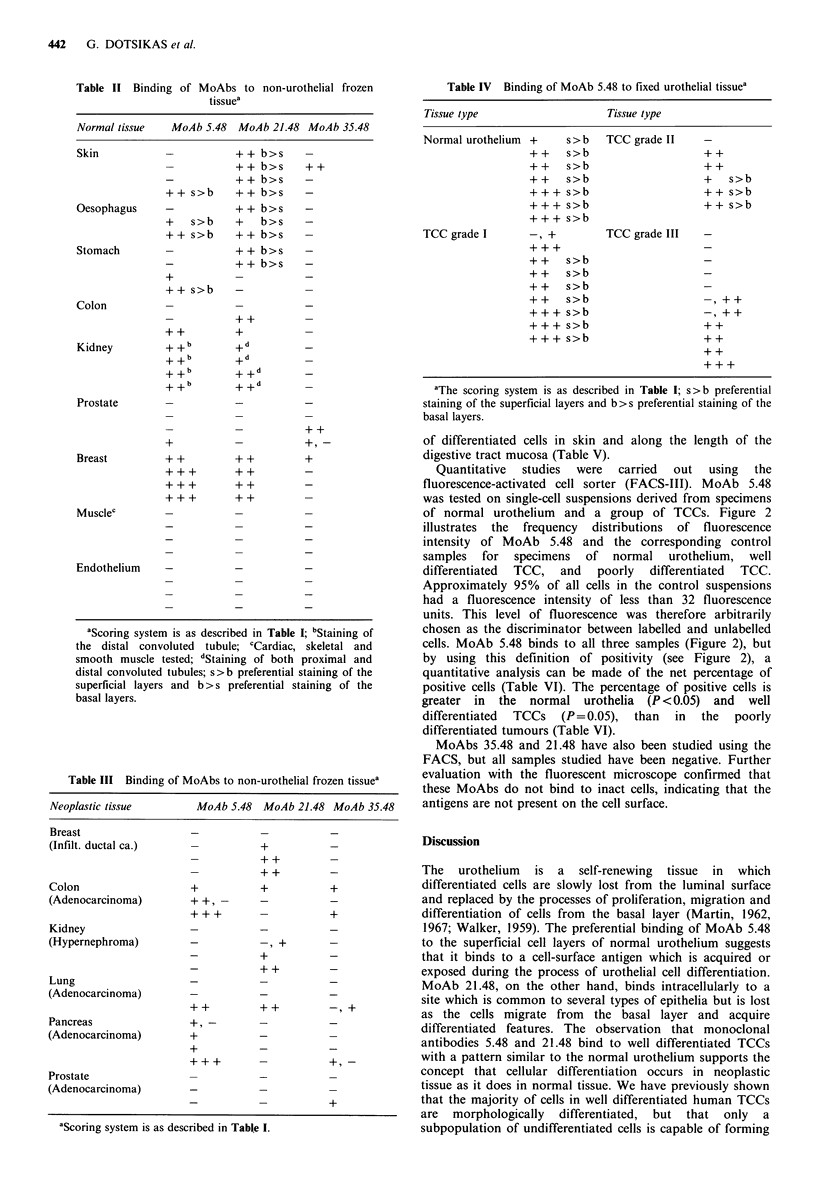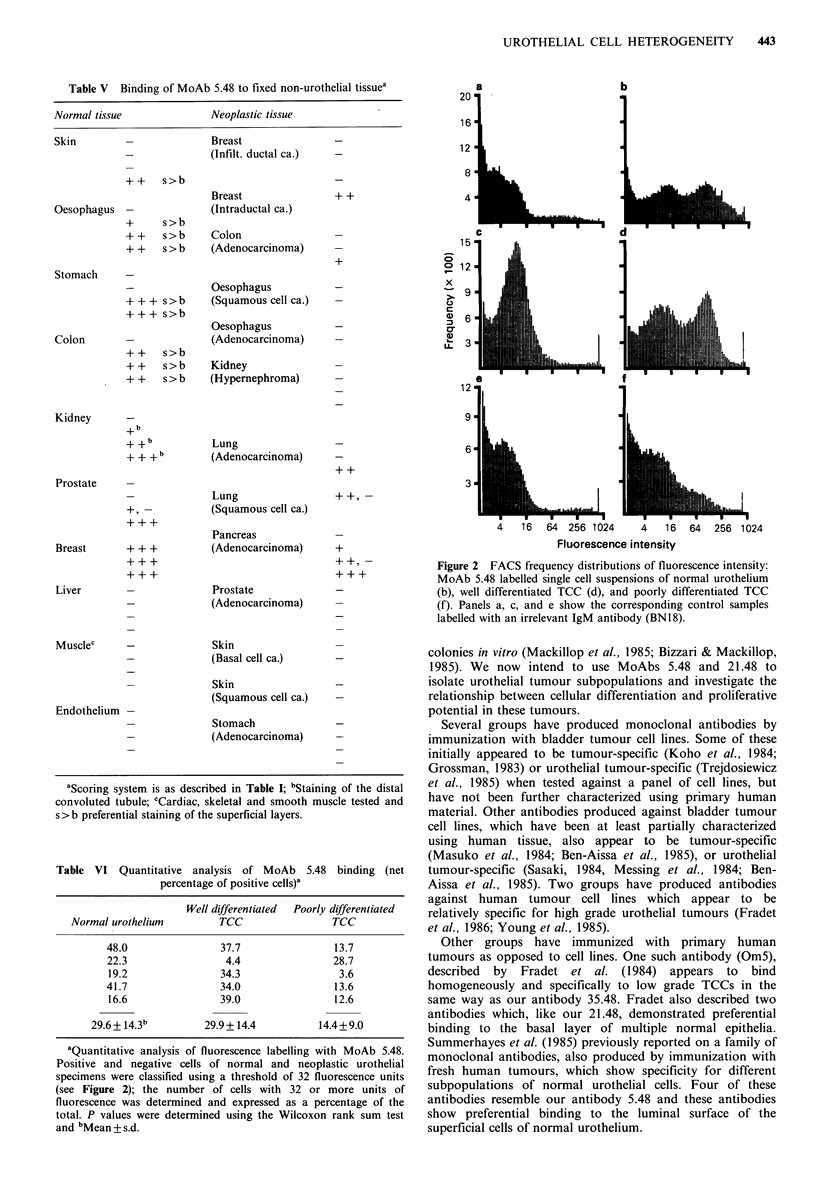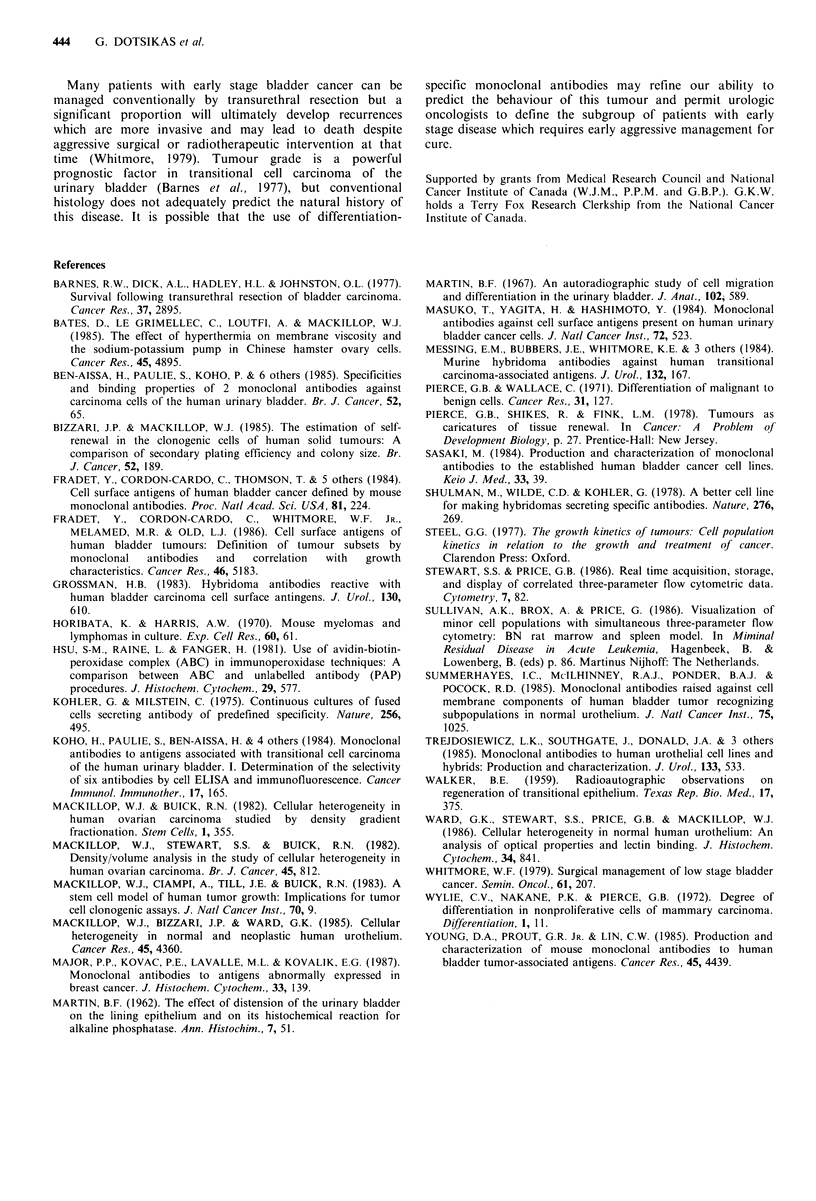# Cellular heterogeneity in normal and neoplastic human urothelium: a study using murine monoclonal antibodies.

**DOI:** 10.1038/bjc.1987.220

**Published:** 1987-10

**Authors:** G. Dotsikas, T. Konowalchuk, P. P. Major, P. E. Kovac, G. K. Ward, S. S. Stewart, G. B. Price, M. M. Elhilali, W. J. Mackillop

**Affiliations:** McGill Cancer Centre, Montreal, Quebec, Canada.

## Abstract

**Images:**


*Br. J. Cancer* (1987), **56**, 439–444 © The Macmillan Press Ltd., 1987

### Cellular heterogeneity in normal and neoplastic human urothelium: A study using murine monoclonal antibodies

G. Dotsikas[1,2], T. Konowalchuk[1,2], P.P. Major[1], P.E. Kovac[1], G.K. Ward[1], S.S. Stewart[1], G.B. Price[1], M.M. Elhilali[1,2] & W.J. Mackillop[1]

[1]*McGill Cancer Centre, Montreal, Quebec and* [2]*Department of Urology, McGill University, Canada*

**Summary** To assist in the description of the cellular heterogeneity present in normal and neoplastic urothelium, a panel of monoclonal antibodies (MoAbs) was raised against human transitional cell carcinoma (TCC) of the urinary bladder. All immunizations were carried out using whole cells and membrane preparations from well differentiated human TCCs. Two fusions produced 145 hybridomas. Following primary screening by ELISA and secondary screening with immunohistochemistry, three useful antibodies were identified. MoAb 35.48 binds to all cell layers of the normal urothelium and well differentiated tumours, but not to the majority of poorly differentiated tumours. MoAb 21.48 binds preferentially to the basal cell layer of normal urothelium and to some well differentiated papillary TCCs, but poorly differentiated tumours exhibit diffusely positive staining. MoAb 21.48 also shows cross-reactivity with basal cell layers of other epithelia. MoAb 5.48 binds preferentially to the superficial cell layers of normal urothelium and well differentiated TCCs, but exhibits less binding in poorly differentiated tumours with loss of the preferential superficial staining. Quantitative flow cytometric studies indicate that MoAb 5.48 binds to a cell-surface antigen which is present on significantly fewer cells of poorly differentiated tumours than on either normal urothelium ($P<0.05$), or well differentiated tumours ($P=0.05$).

The concept of neoplasia as a caricature of the process of tissue renewal was initially proposed by Pierce *et al.* (1978) who demonstrated in animal tumours that terminal differentiation occurs spontaneously and can be induced in several transplantable animal tumours (Pierce & Wallace, 1971; Wylie *et al.*, 1972). It had long been suspected that this might also be true of human tumours (Steel, 1977), but until the development of the *in vitro* clonogenic assay, there was little direct evidence to support this concept. Studies of human ovarian carcinoma using a soft agar colony assay demonstrated that only a small proportion of the total tumour cell population is capable of forming colonies *in culture* (Mackillop & Buick, 1982; Mackillop *et al.*, 1982). Subsequent studies of human urothelium showed similarities between the clonogenic cells of transitional cell carcinomas and the stem cells of the corresponding normal tissue and that morphologically differentiated, non-proliferative cells are numerous in the well differentiated tumours (Mackillop *et al.*, 1985; Bizzari & Mackillop, 1985). These data suggest that terminal differentiation occurs in human transitional cell carcinoma, but the lack of appropriate markers of differentiation makes it difficult to demonstrate that the loss of proliferative potential is linked to the acquisition of features of differentiation. To overcome this difficulty, we have used hybridoma technology (Köhler & Milstein, 1975) to develop a panel of monoclonal antibodies against human transitional cell carcinoma in an attempt to identify markers of differentiation which could be used to study the cellular heterogeneity of human bladder cancer. Fresh human tissue was used for immunizations and for both primary and secondary screening. Tumour cell lines have not been studied because these may be poor models of primary human tumours (Mackillop *et al.*, 1983).

### Materials and methods

### *Clinical material*

Human TCCs were obtained from the operating rooms of the McGill University Teaching Hospitals. Specimens were divided under the supervision of the pathologist with a portion sent for routine histology and a representative sample was made available for laboratory studies. Normal human tissues were obtained from autopsies performed within 6 h of death from any non-neoplastic cause and were confirmed to be normal by light microscopy. Specimens of non-urothelial malignancies were obtained from the tumour bank at the McGill Cancer Centre.

Correspondence: W.J. Mackillop at his present address: Ontario Cancer Foundation, Kingston Regional Cancer Centre, King Street West, Kingston, Ontario K7L 2V7, Canada.
Received 20 May 1987.

### *Membrane preparations*

The procedure for the production of membrane preparations has previously been described by Bates *et al.* (1985).

### *Immunization*

The method used for immunization is essentially as described by Major *et al.* (1987). Briefly, 8 week old BALB/c female mice (Charles River Canada Inc., Constant, Quebec) received i.p. injections of $5\times10^6$ whole tumour cells in incomplete Freund's adjuvant monthly for 5 months. The mice received material from three different well differentiated TCCs. In the seventh month, 300 $\mu$g of the membrane preparation from a fourth well differentiated TCC was injected at two s.c. sites; one week later, three days before fusion, 15 $\mu$g of a fifth membrane preparation was injected intravenously.

### *Cell fusion and hybrid selection*

The technique of cell fusion between immunized mouse spleen cells and the Sp2/0-Ag14 (Sp2) fusion partner (Shulman *et al.*, 1978) and the method of hybrid selection have been described previously by Major *et al.* (1987).

### *Screening of hybridoma supernatants*

*ELISA* Initial screening of hybridoma supernantants was performed using an enzyme-linked immunosorbent assay (ELISA) as described previously (Major *et al.*, 1987). All supernatants were screened against the membrane TCC preparation used for the final boost and against a preparation from normal human liver.

*Avidin/Biotin immunoperoxidase staining* Five-micron frozen and paraffin tissue sections were cut and mounted on gelatin-coated (Tissue Grip, Fisher) and glue-coated glass microscope slides, respectively. Secondary screening of monoclonal antibody supernatants was done using the avidin/biotin immunoperoxidase staining technique as described by Hsu *et al.* (1981). Control sections were labelled with P3 (Horibata & Harris, 1970), an IgG antibody secreted by BALB/c mice myeloma cells.

*Isotype determination*

An ELISA and the Zymed MonoAb-ID EIA kit reagent (Zymed, Burlingame, Ca.) were used following the manufacturer's recommended procedure.

*Indirect immunofluorescence*

Cell suspensions were prepared mechanically, as described previously (Bizzari & Mackillop, 1985). One million cells were suspended in 100 μl monoclonal antibody supernatant and incubated at 4°C for 30 min. Three millilitres of cold alpha-minimal essential medium (Gibco, Burlington, Ontario), supplemented with 10% foetal calf serum (Gibco) and 1% penicillin-streptomycin (30 IU $ml^{-1}$ and 30 μg $ml^{-1}$; Flow Laboratories, Mississuaga, Ontario), were added and the samples were centrifuged for 5 min at 800 *g*. This washing step was repeated twice. The cells were then resuspended in 100 μl fluorescein-conjugated $f(ab')_2$ fragment goat antimouse IgM (Cooper Biomedical, West Chester, Pa.), and incubated at 4°C for 30 min. The cells were washed, and resuspended in PBS for flow cytometry. Control samples were labelled with an irrelevant primary antibody; P3 as an IgG negative control, and BN18 (Sullivan *et al.*, 1986), a mouse monoclonal antibody raised against rat myeloblasts, as an IgM negative control.

*Flow cytometry*

Single-cell suspensions were analysed using a fluorescence-activated cell sorter (FACS-III; Becton-Dickinson, Mountain View, Ca.) linked to a custom-built microcomputer capable of displaying three-parameter data in real time (Stewart & Price, 1986). Fluorescence intensity was measured between 530 and 560 nm for all samples, using an excitation wavelength of 488 nm. The data were displayed as frequency distributions based on the analysis of ~50,000 cells. Fluorescence intensity measurements were standardized as described previously (Ward *et al.*, 1986).

### Results

*Production of hybridomas*

The hybridomas produced from two fusions have been screened and their tissue reactivity characterized. The first fusion produced 85 hybridomas, of which 59 were eliminated due to cross-reactivity with normal liver during the preliminary screening using ELISA. The second fusion resulted in 60 hybridomas, of which 50 were likewise eliminated. The remaining 36 antibodies were then tested against a wide range of normal and neoplastic human frozen tissue sections using the avidin/biotin immunoperoxidase staining technique. Thirty-three MoAbs showed varying degrees of cross-reactivity with other epithelia and were not studied further. Three MoAbs however, initially showed a degree of urothelial specificity and were selected for detailed study on multiple samples of normal and neoplastic human tissue.

*Characteristics and specificities of monoclonal antibodies*

The specificities of the three monoclonal antibodies were initially established using frozen tissue sections.

MoAb 35.48 (IgG,k) was positive on all 6 normal urothelia (Table I) and on 10 of 12 well and moderately differentiated TCCs (grades I and II), but on only 4 of 8 poorly differentiated TCCs (grades III). The diffuse staining

**Table I** Binding of MoAbs to frozen urothelial tissue[a]

| *Tissue type* | *MoAb 5.48* | *MoAb 21.48* | *MoAb 35.48* |
|---|---|---|---|
| Normal urothelium | + + + | + + + b>s | + + + |
| | + s>b | + + | + + + |
| | + + s>b | + + + | + + |
| | + + s>b | + + + | + + |
| | + + s>b | + + | + + |
| | + + s>b | + + b>s | + + + |
| TCC grade I | − | + + | +, − |
| | + | + + b>s | + + |
| | + + | − | − |
| | + + | −, + | +, − |
| | + + | + + | + + |
| | + + + s>b | + + | + + + |
| TCC grade II | + + | − | + |
| | + + | + | + + |
| | + + + | −, + + | − |
| | + + + | + +, − | +, − |
| | + + s>b | −, + + | + + |
| | + + s>b | + + b>s | + + |
| TCC grade III | − | − | − |
| | − | −, + + | + |
| | − | +, − | − |
| | +, − | + + | − |
| | +, − | + +, − | + |
| | +, − | + + + | + + + |
| | + + + | + + + | − |
| | + + + | + + + | +, − |

[a]Avidin/biotin immunoperoxidase staining of tissue sections. + + + = intense positivity, + + = intermediate positivity, + = faint positivity, − = negative. Where varying levels of binding are observed in the same section, two symbols are assigned, the first indicating the dominant staining pattern. s>b preferential staining of the superficial layers. b>s preferential staining of the basal layers.

of the entire thickness of the urothelium with MoAb 35.48 in a well differentiated TCC is shown in panel A of Figure 1 and the corresponding control is shown in panel B. There was faint cross-reactivity with a few of the other normal and neoplastic tissues tested (Table II and III).

MoAb 21.48 (IgG) was positive on multiple normal and malignant tissues (Tables I, II and III). There was a marked preferential binding to the basal layer of some normal urothelial specimens, as shown in Panel E of Figure 1; this binding pattern was also prevalent on a number of other normal epithelia (Table II). A similar pattern was observed in a few of the well differentiated papillary TCCs (Panel F, Figure 1).

MoAb 5.48 (IgM,k) was positive on all normal urothelial specimens tested (Table I) and on 5 of 6 there was preferential staining of the superficial cell layers (Panel C, Figure 1). Staining was most intense along the luminal surface of the superficial layer. Sixteen of 20 TCCs were also positive and in three papillary tumours there was similar preferential staining of the superficial layers (Panel D, Figure 1). There was cross-reactivity with other normal tissues and a few of the unrelated tumours were positive (Tables II and III).

We subsequently studied the binding of the three MoAbs to fixed tissue sections, in which tissue architecture is better preserved allowing for a more detailed evaluation of the staining pattern. We did not find any binding of MoAbs 35.48 and 21.48 to any fixed tissues tested. The antigen bound by MoAb 5.48, however, appeared to survive the fixation process. We observed preferential staining of the superficial cells in all samples of normal urothelium examined and in the majority of well and moderately differentiated TCCs (Table IV). Staining was absent or heterogeneous in many poorly differentiated TCCs, and the preferential staining pattern was not present (Table IV). Further testing of MoAb 5.48 against other human normal and neoplastic fixed tissues revealed its preferential staining

**Figure 1** Avidin/biotin immunoperoxidase staining of normal and neoplastic frozen tissue sections (A, B, E, F) and paraffin sections (C, D), counterstained with haematoxylin. A: well differentiated TCC stained with MoAb 35.48, and B: the corresponding negative control stained with an irrelevant monoclonal antibody (P3). C: normal urothelium stained with MoAb 5.48, D: well differentiated TCC stained with MoAb 5.48, E: normal urothelium stained with MoAb 21.48, and F: well differentiated TCC stained with MoAb 21.48. (All scale bars $= 50\,\mu$m).

**Table II** Binding of MoAbs to non-urothelial frozen tissue[a]

| *Normal tissue* | *MoAb 5.48* | *MoAb 21.48* | *MoAb 35.48* |
|---|---|---|---|
| Skin | – | ++ b>s | – |
| | – | ++ b>s | ++ |
| | – | ++ b>s | – |
| | ++ s>b | ++ b>s | – |
| Oesophagus | – | ++ b>s | – |
| | + s>b | + b>s | – |
| | ++ s>b | ++ b>s | – |
| Stomach | – | ++ b>s | – |
| | – | ++ b>s | – |
| | + | – | – |
| | ++ s>b | – | – |
| Colon | – | – | – |
| | – | ++ | – |
| | ++ | + | – |
| Kidney | ++[b] | +[d] | – |
| | ++[b] | +[d] | – |
| | ++[b] | ++[d] | – |
| | ++[b] | ++[d] | – |
| Prostate | – | – | – |
| | – | – | – |
| | – | – | ++ |
| | + | – | +, – |
| Breast | ++ | ++ | + |
| | +++ | ++ | – |
| | +++ | ++ | – |
| | +++ | ++ | – |
| Muscle[c] | – | – | – |
| | – | – | – |
| | – | – | – |
| | – | – | – |
| Endothelium | – | – | – |
| | – | – | – |
| | – | – | – |
| | – | – | – |

[a]Scoring system is as described in **Table I**; [b]Staining of the distal convoluted tubule; [c]Cardiac, skeletal and smooth muscle tested; [d]Staining of both proximal and distal convoluted tubules; s>b preferential staining of the superficial layers and b>s preferential staining of the basal layers.

**Table III** Binding of MoAbs to non-urothelial frozen tissue[a]

| *Neoplastic tissue* | *MoAb 5.48* | *MoAb 21.48* | *MoAb 35.48* |
|---|---|---|---|
| Breast | – | – | – |
| (Infilt. ductal ca.) | – | + | – |
| | – | ++ | – |
| | – | ++ | – |
| Colon | + | + | + |
| (Adenocarcinoma) | ++, – | – | – |
| | +++ | – | + |
| Kidney | – | – | – |
| (Hypernephroma) | – | –, + | – |
| | – | + | – |
| | – | ++ | – |
| Lung | – | – | – |
| (Adenocarcinoma) | – | – | – |
| | ++ | ++ | –, + |
| Pancreas | +, – | – | – |
| (Adenocarcinoma) | + | – | – |
| | + | – | – |
| | +++ | – | +, – |
| Prostate | – | – | – |
| (Adenocarcinoma) | – | – | – |
| | – | – | + |

[a]Scoring system is as described in **Table I**.

**Table IV** Binding of MoAb 5.48 to fixed urothelial tissue[a]

| *Tissue type* | | *Tissue type* | |
|---|---|---|---|
| Normal urothelium | + s>b | TCC grade II | – |
| | ++ s>b | | ++ |
| | ++ s>b | | ++ |
| | ++ s>b | | + s>b |
| | +++ s>b | | ++ s>b |
| | +++ s>b | | ++ s>b |
| | +++ s>b | | |
| TCC grade I | –, + | TCC grade III | – |
| | +++ | | – |
| | ++ s>b | | – |
| | ++ s>b | | – |
| | ++ s>b | | – |
| | ++ s>b | | –, ++ |
| | +++ s>b | | –, ++ |
| | +++ s>b | | ++ |
| | +++ s>b | | ++ |
| | | | ++ |
| | | | +++ |

[a]The scoring system is as described in **Table I**; s>b preferential staining of the superficial layers and b>s preferential staining of the basal layers.

of differentiated cells in skin and along the length of the digestive tract mucosa (Table V).

Quantitative studies were carried out using the fluorescence-activated cell sorter (FACS-III). MoAb 5.48 was tested on single-cell suspensions derived from specimens of normal urothelium and a group of TCCs. Figure 2 illustrates the frequency distributions of fluorescence intensity of MoAb 5.48 and the corresponding control samples for specimens of normal urothelium, well differentiated TCC, and poorly differentiated TCC. Approximately 95% of all cells in the control suspensions had a fluorescence intensity of less than 32 fluorescence units. This level of fluorescence was therefore arbitrarily chosen as the discriminator between labelled and unlabelled cells. MoAb 5.48 binds to all three samples (Figure 2), but by using this definition of positivity (see Figure 2), a quantitative analysis can be made of the net percentage of positive cells (Table VI). The percentage of positive cells is greater in the normal urothelia ($P<0.05$) and well differentiated TCCs ($P=0.05$), than in the poorly differentiated tumours (Table VI).

MoAbs 35.48 and 21.48 have also been studied using the FACS, but all samples studied have been negative. Further evaluation with the fluorescent microscope confirmed that these MoAbs do not bind to inact cells, indicating that the antigens are not present on the cell surface.

### Discussion

The urothelium is a self-renewing tissue in which differentiated cells are slowly lost from the luminal surface and replaced by the processes of proliferation, migration and differentiation of cells from the basal layer (Martin, 1962, 1967; Walker, 1959). The preferential binding of MoAb 5.48 to the superficial cell layers of normal urothelium suggests that it binds to a cell-surface antigen which is acquired or exposed during the process of urothelial cell differentiation. MoAb 21.48, on the other hand, binds intracellularly to a site which is common to several types of epithelia but is lost as the cells migrate from the basal layer and acquire differentiated features. The observation that monoclonal antibodies 5.48 and 21.48 bind to well differentiated TCCs with a pattern similar to the normal urothelium supports the concept that cellular differentiation occurs in neoplastic tissue as it does in normal tissue. We have previously shown that the majority of cells in well differentiated human TCCs are morphologically differentiated, but that only a subpopulation of undifferentiated cells is capable of forming

**Table V** Binding of MoAb 5.48 to fixed non-urothelial tissue[a]

| *Normal tissue* | | | *Neoplastic tissue* | |
|---|---|---|---|---|
| Skin | – | | Breast | – |
| | – | | (Infilt. ductal ca.) | – |
| | – | | | |
| | ++ | s>b | | – |
| | | | Breast | ++ |
| Oesophagus | – | | (Intraductal ca.) | |
| | + | s>b | | |
| | ++ | s>b | Colon | – |
| | ++ | s>b | (Adenocarcinoma) | – |
| | | | | + |
| Stomach | – | | | |
| | – | | Oesophagus | – |
| | +++ | s>b | (Squamous cell ca.) | – |
| | +++ | s>b | | |
| | | | Oesophagus | – |
| Colon | – | | (Adenocarcinoma) | – |
| | ++ | s>b | | |
| | ++ | s>b | Kidney | – |
| | ++ | s>b | (Hypernephroma) | – |
| | | | | – |
| | | | | – |
| Kidney | – | | | |
| | +[b] | | | |
| | ++[b] | | Lung | – |
| | +++[b] | | (Adenocarcinoma) | – |
| | | | | ++ |
| Prostate | – | | | |
| | – | | Lung | ++, – |
| | +, – | | (Squamous cell ca.) | |
| | +++ | | | |
| | | | Pancreas | – |
| Breast | +++ | | (Adenocarcinoma) | + |
| | +++ | | | ++, – |
| | +++ | | | +++ |
| Liver | – | | Prostate | – |
| | – | | (Adenocarcinoma) | – |
| | – | | | – |
| | – | | | – |
| Muscle[c] | – | | Skin | – |
| | – | | (Basal cell ca.) | – |
| | – | | | |
| | – | | Skin | – |
| | | | (Squamous cell ca.) | – |
| Endothelium | – | | | |
| | – | | Stomach | – |
| | – | | (Adenocarcinoma) | – |
| | – | | | – |
| | | | | – |

[a]Scoring system is as described in **Table I**; [b]Staining of the distal convoluted tubule; [c]Cardiac, skeletal and smooth muscle tested and s>b preferential staining of the superficial layers.

**Table VI** Quantitative analysis of MoAb 5.48 binding (net percentage of positive cells)[a]

| *Normal urothelium* | *Well differentiated TCC* | *Poorly differentiated TCC* |
|---|---|---|
| 48.0 | 37.7 | 13.7 |
| 22.3 | 4.4 | 28.7 |
| 19.2 | 34.3 | 3.6 |
| 41.7 | 34.0 | 13.6 |
| 16.6 | 39.0 | 12.6 |
| $29.6 \pm 14.3$[b] | $29.9 \pm 14.4$ | $14.4 \pm 9.0$ |

[a]Quantitative analysis of fluorescence labelling with MoAb 5.48. Positive and negative cells of normal and neoplastic urothelial specimens were classified using a threshold of 32 fluorescence units (see **Figure 2**); the number of cells with 32 or more units of fluorescence was determined and expressed as a percentage of the total. *P* values were determined using the Wilcoxon rank sum test and [b]Mean ± s.d.

**Figure 2** FACS frequency distributions of fluorescence intensity: MoAb 5.48 labelled single cell suspensions of normal urothelium (b), well differentiated TCC (d), and poorly differentiated TCC (f). Panels a, c, and e show the corresponding control samples labelled with an irrelevant IgM antibody (BN18).

colonies *in vitro* (Mackillop *et al.*, 1985; Bizzari & Mackillop, 1985). We now intend to use MoAbs 5.48 and 21.48 to isolate urothelial tumour subpopulations and investigate the relationship between cellular differentiation and proliferative potential in these tumours.

Several groups have produced monoclonal antibodies by immunization with bladder tumour cell lines. Some of these initially appeared to be tumour-specific (Koho *et al.*, 1984; Grossman, 1983) or urothelial tumour-specific (Trejdosiewicz *et al.*, 1985) when tested against a panel of cell lines, but have not been further characterized using primary human material. Other antibodies produced against bladder tumour cell lines, which have been at least partially characterized using human tissue, also appear to be tumour-specific (Masuko *et al.*, 1984; Ben-Aissa *et al.*, 1985), or urothelial tumour-specific (Sasaki, 1984, Messing *et al.*, 1984; Ben-Aissa *et al.*, 1985). Two groups have produced antibodies against human tumour cell lines which appear to be relatively specific for high grade urothelial tumours (Fradet *et al.*, 1986; Young *et al.*, 1985).

Other groups have immunized with primary human tumours as opposed to cell lines. One such antibody (Om5), described by Fradet *et al.* (1984) appears to bind homogeneously and specifically to low grade TCCs in the same way as our antibody 35.48. Fradet also described two antibodies which, like our 21.48, demonstrated preferential binding to the basal layer of multiple normal epithelia. Summerhayes *et al.* (1985) previously reported on a family of monoclonal antibodies, also produced by immunization with fresh human tumours, which show specificity for different subpopulations of normal urothelial cells. Four of these antibodies resemble our antibody 5.48 and these antibodies show preferential binding to the luminal surface of the superficial cells of normal urothelium.

Many patients with early stage bladder cancer can be managed conventionally by transurethral resection but a significant proportion will ultimately develop recurrences which are more invasive and may lead to death despite aggressive surgical or radiotherapeutic intervention at that time (Whitmore, 1979). Tumour grade is a powerful prognostic factor in transitional cell carcinoma of the urinary bladder (Barnes *et al.*, 1977), but conventional histology does not adequately predict the natural history of this disease. It is possible that the use of differentiation-specific monoclonal antibodies may refine our ability to predict the behaviour of this tumour and permit urologic oncologists to define the subgroup of patients with early stage disease which requires early aggressive management for cure.

Supported by grants from Medical Research Council and National Cancer Institute of Canada (W.J.M., P.P.M. and G.B.P.). G.K.W. holds a Terry Fox Research Clerkship from the National Cancer Institute of Canada.

### References

BARNES, R.W., DICK, A.L., HADLEY, H.L. & JOHNSTON, O.L. (1977). Survival following transurethral resection of bladder carcinoma. *Cancer Res.*, **37,** 2895.

BATES, D., LE GRIMELLEC, C., LOUTFI, A. & MACKILLOP, W.J. (1985). The effect of hyperthermia on membrane viscosity and the sodium-potassium pump in Chinese hamster ovary cells. *Cancer Res.*, **45,** 4895.

BEN-AISSA, H., PAULIE, S., KOHO, P. & 6 others (1985). Specificities and binding properties of 2 monoclonal antibodies against carcinoma cells of the human urinary bladder. *Br. J. Cancer,* **52,** 65.

BIZZARI, J.P. & MACKILLOP, W.J. (1985). The estimation of self-renewal in the clonogenic cells of human solid tumours: A comparison of secondary plating efficiency and colony size. *Br. J. Cancer,* **52,** 189.

FRADET, Y., CORDON-CARDO, C., THOMSON, T. & 5 others (1984). Cell surface antigens of human bladder cancer defined by mouse monoclonal antibodies. *Proc. Natl Acad. Sci. USA,* **81,** 224.

FRADET, Y., CORDON-CARDO, C., WHITMORE, W.F. JR., MELAMED, M.R. & OLD, L.J. (1986). Cell surface antigens of human bladder tumours: Definition of tumour subsets by monoclonal antibodies and correlation with growth characteristics. *Cancer Res.*, **46,** 5183.

GROSSMAN, H.B. (1983). Hybridoma antibodies reactive with human bladder carcinoma cell surface antingens. *J. Urol.*, **130,** 610.

HORIBATA, K. & HARRIS, A.W. (1970). Mouse myelomas and lymphomas in culture. *Exp. Cell Res.*, **60,** 61.

HSU, S-M., RAINE, L. & FANGER, H. (1981). Use of avidin-biotin-peroxidase complex (ABC) in immunoperoxidase techniques: A comparison between ABC and unlabelled antibody (PAP) procedures. *J. Histochem. Cytochem.*, **29,** 577.

KOHLER, G. & MILSTEIN, C. (1975). Continuous cultures of fused cells secreting antibody of predefined specificity. *Nature,* **256,** 495.

KOHO, H., PAULIE, S., BEN-AISSA, H. & 4 others (1984). Monoclonal antibodies to antigens associated with transitional cell carcinoma of the human urinary bladder. I. Determination of the selectivity of six antibodies by cell ELISA and immunofluorescence. *Cancer Immunol. Immunother.*, **17,** 165.

MACKILLOP, W.J. & BUICK, R.N. (1982). Cellular heterogeneity in human ovarian carcinoma studied by density gradient fractionation. *Stem Cells,* **1,** 355.

MACKILLOP, W.J., STEWART, S.S. & BUICK, R.N. (1982). Density/volume analysis in the study of cellular heterogeneity in human ovarian carcinoma. *Br. J. Cancer,* **45,** 812.

MACKILLOP, W.J., CIAMPI, A., TILL, J.E. & BUICK, R.N. (1983). A stem cell model of human tumor growth: Implications for tumor cell clonogenic assays. *J. Natl Cancer Inst.*, **70,** 9.

MACKILLOP, W.J., BIZZARI, J.P. & WARD, G.K. (1985). Cellular heterogeneity in normal and neoplastic human urothelium. *Cancer Res.*, **45,** 4360.

MAJOR, P.P., KOVAC, P.E., LAVALLE, M.L. & KOVALIK, E.G. (1987). Monoclonal antibodies to antigens abnormally expressed in breast cancer. *J. Histochem. Cytochem.*, **33,** 139.

MARTIN, B.F. (1962). The effect of distension of the urinary bladder on the lining epithelium and on its histochemical reaction for alkaline phosphatase. *Ann. Histochim.*, **7,** 51.

MARTIN, B.F. (1967). An autoradiographic study of cell migration and differentiation in the urinary bladder. *J. Anat.*, **102;** 589.

MASUKO, T., YAGITA, H. & HASHIMOTO, Y. (1984). Monoclonal antibodies against cell surface antigens present on human urinary bladder cancer cells. *J. Natl Cancer Inst.*, **72,** 523.

MESSING, E.M., BUBBERS, J.E., WHITMORE, K.E. & 3 others (1984). Murine hybridoma antibodies against human transitional carcinoma-associated antigens. *J. Urol.*, **132,** 167.

PIERCE, G.B. & WALLACE, C. (1971). Differentiation of malignant to benign cells. *Cancer Res.*, **31,** 127.

PIERCE, G.B., SHIKES, R. & FINK, L.M. (1978). Tumours as caricatures of tissue renewal. In *Cancer: A Problem of Development Biology,* p. 27. Prentice-Hall: New Jersey.

SASAKI, M. (1984). Production and characterization of monoclonal antibodies to the established human bladder cancer cell lines. *Keio J. Med.*, **33,** 39.

SHULMAN, M., WILDE, C.D. & KOHLER, G. (1978). A better cell line for making hybridomas secreting specific antibodies. *Nature,* **276,** 269.

STEEL, G.G. (1977). *The growth kinetics of tumours: Cell population kinetics in relation to the growth and treatment of cancer.* Clarendon Press: Oxford.

STEWART, S.S. & PRICE, G.B. (1986). Real time acquisition, storage, and display of correlated three-parameter flow cytometric data. *Cytometry,* **7,** 82.

SULLIVAN, A.K., BROX, A. & PRICE, G. (1986). Visualization of minor cell populations with simultaneous three-parameter flow cytometry: BN rat marrow and spleen model. In *Miminal Residual Disease in Acute Leukemia,* Hagenbeek, B. & Lowenberg, B. (eds) p. 86. Martinus Nijhoff: The Netherlands.

SUMMERHAYES, I.C., McILHINNEY, R.A.J., PONDER, B.A.J. & POCOCK, R.D. (1985). Monoclonal antibodies raised against cell membrane components of human bladder tumor recognizing subpopulations in normal urothelium. *J. Natl Cancer Inst.*, **75,** 1025.

TREJDOSIEWICZ, L.K., SOUTHGATE, J., DONALD, J.A. & 3 others (1985). Monoclonal antibodies to human urothelial cell lines and hybrids: Production and characterization. *J. Urol.*, **133,** 533.

WALKER, B.E. (1959). Radioautographic observations on regeneration of transitional epithelium. *Texas Rep. Bio. Med.*, **17,** 375.

WARD, G.K., STEWART, S.S., PRICE, G.B. & MACKILLOP, W.J. (1986). Cellular heterogeneity in normal human urothelium: An analysis of optical properties and lectin binding. *J. Histochem. Cytochem.*, **34,** 841.

WHITMORE, W.F. (1979). Surgical management of low stage bladder cancer. *Semin. Oncol.*, **61,** 207.

WYLIE, C.V., NAKANE, P.K. & PIERCE, G.B. (1972). Degree of differentiation in nonproliferative cells of mammary carcinoma. *Differentiation,* **1,** 11.

YOUNG, D.A., PROUT, G.R. JR. & LIN, C.W. (1985). Production and characterization of mouse monoclonal antibodies to human bladder tumor-associated antigens. *Cancer Res.*, **45,** 4439.